# SCTN: Event-based object tracking with energy-efficient deep convolutional spiking neural networks

**DOI:** 10.3389/fnins.2023.1123698

**Published:** 2023-02-16

**Authors:** Mingcheng Ji, Ziling Wang, Rui Yan, Qingjie Liu, Shu Xu, Huajin Tang

**Affiliations:** ^1^College of Computer Science and Technology, Zhejiang University, Hangzhou, China; ^2^College of Computer Science, Zhejiang University of Technology, Hangzhou, China; ^3^Machine Intelligence Laboratory, China Nanhu Academy of Electronics and Information Technology, Jiaxing, China; ^4^Zhejiang Lab, Hangzhou, China

**Keywords:** spiking neural networks, event cameras, object tracking, exponential IoU, event-based tracking dataset

## Abstract

Event cameras are asynchronous and neuromorphically inspired visual sensors, which have shown great potential in object tracking because they can easily detect moving objects. Since event cameras output discrete events, they are inherently suitable to coordinate with Spiking Neural Network (SNN), which has a unique event-driven computation characteristic and energy-efficient computing. In this paper, we tackle the problem of event-based object tracking by a novel architecture with a discriminatively trained SNN, called the Spiking Convolutional Tracking Network (SCTN). Taking a segment of events as input, SCTN not only better exploits implicit associations among events rather than event-wise processing, but also fully utilizes precise temporal information and maintains the sparse representation in segments instead of frames. To make SCTN more suitable for object tracking, we propose a new loss function that introduces an exponential Intersection over Union (IoU) in the voltage domain. To the best of our knowledge, this is the first tracking network directly trained with SNN. Besides, we present a new event-based tracking dataset, dubbed DVSOT21. In contrast to other competing trackers, experimental results on DVSOT21 demonstrate that our method achieves competitive performance with very low energy consumption compared to ANN based trackers with very low energy consumption compared to ANN based trackers. With lower energy consumption, tracking on neuromorphic hardware will reveal its advantage.

## 1. Introduction

Object tracking is a nontrivial problem in computer vision, and is widely used in security monitoring, sports events broadcasting, robotics, unmanned aerial vehicles and other fields. In recent years, object tracking with traditional cameras has become very mature, represented by algorithms based on Siamese networks (Zhang et al., 2020, 2021) and Transformers (Chen et al., 2021; Wang et al., 2021; Cui et al., 2022). Unfortunately, traditional cameras have difficulty capturing moving objects under extreme conditions of high speed and high dynamic range.

Event camera, such as the Dynamic Vision Sensor (DVS), is a novel, asynchronous, and neuromorphically inspired visual sensor (Gallego et al., 2020). Each pixel on the sensor can independently detect the illumination changes in the scene, and once the changes exceed the threshold, it will output visual information in the form of events. Since the brightness changes are usually caused by the movement of objects, event cameras only capture the dynamic information from their visual input and output it in the form of events, ignoring the static information in the scene (Khoei et al., 2019).

In contrast to traditional cameras, event cameras have the advantages of high time resolution, high dynamic range, low power consumption, and low information redundancy. Therefore, it can well capture the movement of objects in the dark environment or the fast-moving scene without motion blur, which is ideal for object tracking. Several event-based object tracking methods have been proposed in the past few years. which can be roughly divided into two categories. The first category is that each incoming event is determined in time whether it belongs to the target or the background. In (Litzenberger et al., 2006), the authors performed event-based object tracking with the clustering algorithm, where each incoming event is assigned to a cluster and then the parameters of the cluster are updated. Ni et al. (2015) proposed an event-based tracking method by making a continuous and iterative estimation of the geometric transformation. Although these methods are very fast to track, they are easily affected by noise events. A single noise event may cause the tracker to make a wrong inference. Furthermore, they are susceptible to complex background, shape variation and so on. It is difficult to decide whether it belongs to the target based on a single event. Because they cannot utilize the implicit associations between events, which mean the temporal and spatial associations between events.

The other category is to collect events over a period of time and track objects according to their features. In Lagorce et al. (2014), the authors proposed an asynchronous event-based multi-kernel algorithm, which is based on the assumption that events generated by object motion approximately follow a bivariate normal distribution. In Mitrokhin et al. (2018), the authors presented a tracking-by-detection method, where a novel time-image representation was proposed. This representation gives temporal information to events projected to the same pixel, which facilitates subsequent motion compensation. RMRNet (Chen et al., 2020) was formulated to predict 5-DoF object motion regression, which allows end-to-end event-based object tracking. These methods will have a certain delay compared to the first category, but usually they will make the tracking more accurate.

In addition to these methods, traditional trackers applied in frame-based video sequences can also be used for event-based tracking. In this way, the event stream is expected to be converted into frames at first. In Henriques et al. (2014), the authors proposed kernelized correlation filters (KCF), using multi-channel features and mapping the ridge regression of linear space to nonlinear space through the kernel function, and the Fourier space diagonalization is used in the circulant matrices. Siamese network and its variants have achieved excellent performance in recent years. SiamFC (Bertinetto et al., 2016) is the pioneering work, which uses a fully convolutional Siamese network for object tracking, and the frame rate exceeds the real-time requirements. Inspired by this work, many algorithms based on Siamese networks were generated (Li et al., 2018; Wang et al., 2019; Zhang et al., 2020, 2021), all achieving very good performance in object tracking. On the basis of Siamese networks, TrDiMP (Wang et al., 2021) combines Transformer and exploits temporal context for object tracking. The transformer encoder facilitates object templates through attention-based feature enhancement, which is beneficial for the generation of high quality tracking models. The transformer decoder propagates tracking cues from previous templates to the current frame, thus simplifying the object search process.

However, the event stream should be firstly converted into static images when ANNs are used to process the output of the event cameras, leading to the loss of precise temporal information within events. Since events contain precise spatio-temporal information, they are more suitable to be processed by SNN, which uses spike coding to integrate timing information (Ghosh-Dastidar and Adeli, 2009). In this way, events are treated as spikes that can be handled directly by SNN (Jiang et al., 2021). SiamSNN (Luo et al., 2021), the deep SNN for object tracking, uses the model converted from SiamFC and achieves low precision loss on the benchmarks. But SiamSNN is not directly trained with SNN, it is trained using the conversion algorithm with pretrained ANN.

In this work, we propose a novel tracking architecture, referred to as Spiking Convolutional Tracking Network (SCTN), for single object tracking in event-based video sequences. SCTN can not only process the event stream without any additional operations, but also make full use of the temporal information in it. Unlike Nam and Han (2016), online learning is dispensable in our model, since it is time-consuming during test and it contributes little to tracking performance. The power of this online learning method stems from fine-tuning the network according to the tracking results in the first few testing frames, however, the network will be updated in the wrong direction due to taking the inaccurate tracking results as online training samples.

As far as we know, SCTN is the first event based single object tracking network directly trained with SNN. Compared with ANN-based tracking methods, our method can accept the input of event stream without any preprocessing operations and take full advantage of the temporal information in it, and especially show remarkable capabilities of energy-efficient computing. We propose a new loss function that introduces an exponential IoU between ground-truths and training bounding boxes in the voltage domain, while the candidate bounding box corresponding to the largest voltage is regarded as the target bounding box in the test. Besides, we present a novel publicly available event-based tracking dataset, named DVSOT21, under challenge conditions by implementing the bounding boxes generation module to extend the ESIM simulator (Rebecq et al., 2018).

## 2. Materials and methods

In this section, we first present the description of events and spiking neuron model used in SCTN in Section 2.1 and Section 2.2. Then we describe the network architecture and tracking process in section 2.3. Samples generation for training, fine-tuning and target bounding box selection will be shown in Section 2.4. We introduce the learning algorithm and loss function in Section 2.5. Finally, the target bounding box selection will be demonstrated in Section 2.6.

### 2.1. Events description

**Figure 1:**
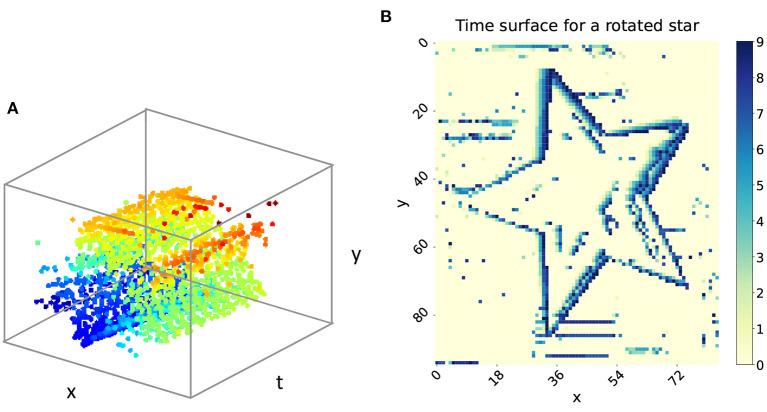
**(A)** The 3D visualization of the rotated star. **(B)** The time surface of the rotated star.

Each of the events can be described as a quadruple (*x, y, t, p*), where (*x, y*) denotes the position of the triggered event, *t* represents the timestamp, and the polarity *p* = +1 means the increasing brightness while *p* = −1 means the decreasing brightness. The 3D visualization of the event stream is illustrated in Figure 1A, which indicates a rotated star. Figure 1B shows the time surface of the rotated star, where the color from yellow to blue represents the time trajectory from old to new events. From this we can deduce that the star is moving clockwise.

### 2.2. Spiking neuron model

Here, the current-based leaky integrate-and-fire (C-LIF) neuron model (Gütig, 2016) is used as the basic computational unit in SCTN. Assuming there are N afferent neurons, the voltage of the C-LIF spiking neuron can be calculated as:

(1) $$\begin{array}{l} V_{t}=L_{t}-E_{t} \end{array}$$

(2) $$\begin{array}{l} L_{t}=\overset{N}{\underset{i=1}{\sum }}w_{i}\underset{t_{i}^{j}<t}{\sum }K\left(t-t_{i}^{j}\right) \end{array}$$

(3) $$\begin{array}{l} E_{t}=\vartheta \underset{t_{s}^{j}<t}{\sum }\exp\left(-\frac{t-t_{s}^{j}}{\alpha_{m}}\right) \end{array}$$

(4) $$\begin{array}{l} K\left(t-t_{i}^{j}\right)=V_{0}\left[\exp\left(-\frac{t-t_{i}^{j}}{\alpha_{m}}\right)-\exp\left(-\frac{t-t_{i}^{j}}{\alpha_{s}}\right)\right] \end{array}$$

where *w*_*i*_ is the synaptic efficacy, $t_{i}^{j}$ denotes the time of the j-th input spike from the i-th afferent neuron, and $t_{s}^{j}$ denotes the time of the j-th firing spike. Each spike at time $t_{i}^{j}$ contributes a postsynaptic potential (PSP), whose shape is determined by the double exponential kernel function $K\left(t-t_{i}^{j}\right)$. *V*_0_ is a normalization factor that normalizes the maximum value of the kernel to 1. α_*m*_, α_*s*_ mean the time decay factors, which are learnable parameters in SCTN. ϑ denotes the threshold of the neuron and it is equal to 0.5 in our experiments. *L*_*t*_ is the dynamics of the leaky integrate-and-fire (LIF) neuron model (Gerstner and Kistler, 2002), which describes the input synaptic current from N presynaptic neurons. Compared with the LIF model, the C-LIF model has one more reset item *E*_*t*_, indicating that each output spike will suppress the voltage for a moment. For C-LIF model, a spike is triggered when *V*_*t*_ exceeds ϑ and then *V*_*t*_ is reset.

### 2.3. Network architecture and tracking process

**Figure 2:**
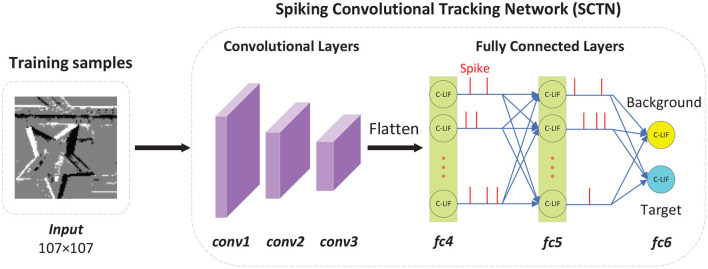
The architecture of spiking convolutional tracking network. In this figure, the intensity images are reconstructed from all the events within 10ms. Note that the network architecture of SCTN is relatively simple, including three convolutional layers and three fully-connected layers. There are only two neurons in the last fully connected layer, representing background and target respectively.

The architecture of SCTN is illustrated in Figure 2, where the intensity images are the visualization of all the events within 10 ms. Too small a time window will make the accumulated events sparse and too large a time window may result in the motion blur. The experiments show that 10ms is a compromise value. So in our work, a segment is defined as all the events accumulated within 10 ms, which can be processed by SNN without any preprocessing, rather than being processed as converted into a frame.

Our approach consists of two phases: training and test, where the sample generator plays an important role. During training, the input samples produced by sample generator are used for training SCTN, which contains three convolutional layers and three fully-connected layers. In the first convolutional layer, the size of the input image is determined by the largest bounding box, which is 107 × 107 in this paper. For the bounding boxes smaller than 107 × 107, only the neurons corresponding to the events within the bounding box will emit spikes, otherwise they are set to no output spikes. There are only two neurons in the last fully connected layer, one indicates background and the other indicates target. In addition, we apply adaptive learnable parameters to all the C-LIF neurons, which are beneficial to improve tracking performance.

**Figure 3:**
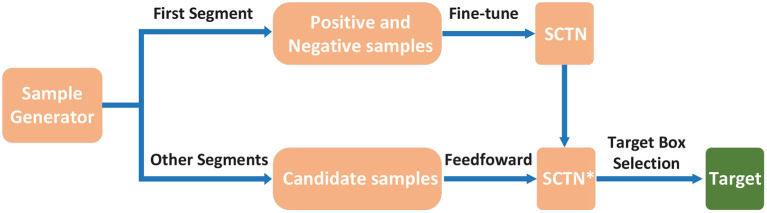
Test flowchart of SCTN. In the test phase, positive and negative samples are generated by the sample generator in order to fine-tune SCTN using the first segment of the test sequence. For other segments, the candidate samples are also created by the sample generator, in which we need to choose an optimal candidate sample as the target bounding box through the evaluation of SCTN^*^.

As shown in Figure 3, the sample generator is responsible for generating fine-tuning samples and candidate samples in the process of tracking by SCTN. The details can be found in Section 2.4. In the first segment of the test sequence, the positive and negative samples are generated for fine-tuning SCTN so that the target features specific to each test sequence can be obtained. To trace the target in the next segments, candidate samples should be produced by the sample generator. Then SCTN^*^ will choose an optimal candidate sample as the estimated target bounding box.

### 2.4. Samples generation

**Figure 4:**
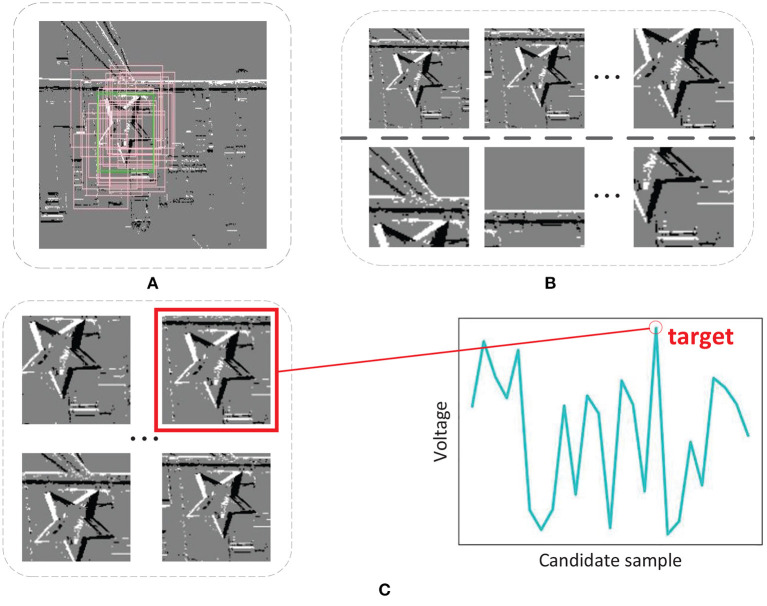
**(A)** Sample generator. **(B)** Positive and negative samples. **(C)** Candidate samples and target bounding box selection.

Owing to the lack of training samples, we need to utilize the original data to generate more samples. In this paper, we adopt the sample generator inspired by Nam and Han (2016). As illustrated in Figure 4B, 20 positive and 40 negative samples are generated based on uniform distribution near the ground-truths from every segment during training, where positive and negative samples have ≥0.7 and ≤0.5 IoU overlap ratios with ground-truths. Note that this number of samples has been able to meet the training requirements. Increasing the number of samples may lead to overfitting of the network, because these generated training samples have certain similarities.

Similarly for fine-tuning, we collect 500 positive and 2,000 negative samples in the first segment of a test sequence and the limitation of IoU is the same as the ones in training. The difference from training is that 500 positive samples are collected based on normal distribution, 1,000 negative samples are collected based on uniform distribution and another 1,000 negative samples are collected within the whole image. In this way, the features of the whole image can be extracted to train SCTN, making SCTN more discriminant to the target and background.

In every segment except the first segment during the test phase, we choose 256 target candidates generated based on normal distribution near the estimated target bounding box in the previous segment, which are displayed in Figure 4C.

### 2.5. Learning algorithm and loss function

The learning algorithm used in SCTN is Spatio-Temporal Credit Assignment (STCA) (Gu et al., 2019), which is a supervised learning algorithm for training deep SNN with the output of multi-spike. STCA introduces an iterative strategy for backpropagating the residual error in the spatio-temporal domain based on C-LIF spiking neuron model. The details of STCA algorithm can be seen in Gu et al. (2019).

In our work, to better apply SCTN to object tracking, we propose a new loss function that introduces an exponential IoU in the voltage domain, making SCTN more sensitive to well-classified positive samples. Note that well-classified positive samples mean they have large IoUs with ground-truths. The error signal to be backpropagated is the difference between the expected and the actual output voltage of the last layer in SCTN. So we can define the loss function as follow:

(5) $$\begin{array}{l} LOSS=\{\begin{array}{ll} \vartheta +R_{s}-V_{max}, & fail\;to\;fire\\ V_{max}-R_{s}-\vartheta , & fire\;wrongly \end{array} \end{array}$$

where *V*_*max*_ denotes the maximum voltage of the output neuron over all time steps, *R*_*s*_ represents the feedback of a sample to the voltage and *R*_*s*_ + ϑ means the expected output voltage. Assuming the IoU overlap ratios between the training bounding boxes and the ground-truths are *O*_*s*_, where s denotes a positive sample or a negative sample, the feedback of a sample can be calculated using the following function:

(6) $$\begin{array}{l} R_{s}=\{\begin{array}{ll} \frac{e^{\beta O_{s}}}{\gamma }, & s\in positive\;samples\\ -0.3, & s\in negative\;samples \end{array} \end{array}$$

where β = 5 and γ = 100, mapping the values of positive feedback between (0.3, 1.5). Additionally, the exponential form allows positive samples with larger IoU to get higher positive feedback.

### 2.6. Target bounding box selection

During test, a sequence is presented and the target location is only given in the first segment. In other words, we only know the ground-truth of the target in the first segment and we are expected to figure out the target locations in the next segments.

Suppose we want to find out the bounding box of the target in the i-th segment, 256 target candidates *C*^1^, …, *C*^256^ are sampled around the estimated target bounding box in the (i-1)-th segment and they are evaluated using SCTN. Then we obtain 256 scores, *s*(*C*^*k*^), *k* = 1, …, 256, for each candidate. As shown in Figure 4C, the optimal target bounding box C^*^ is found with the maximum score as below:

(7) $$\begin{array}{l} C^{*}=\underset{C^{k}}{\arg\max s\left(C^{k}\right)} \end{array}$$

The test procedure of tracking with SCTN can be summarized in Algorithm 1.

**Algorithm 1:** The test procedure of tracking with SCTN.

**Input:** test sequence *S*, ground-truth *G*_1_ in *S*_1_, trained SCTN model *M*.
**Output:** target bounding box *T*_*i*_ in *S*.
1: Generate positive and negative samples around *G*_1_
2: The generated samples are used to fine-tune *M*
3: Set *T*_1_ = *G*_1_
4: Initialize the translation range employed in generating target candidates
5: **for** *i* ← 2 **to** nFrames **do**
6: Generate target candidates *C* around *T*_*i*−1_
7: Feed *C* to *M*
8: Obtain the target bounding boxes corresponding to the top 3 maximum voltage values through the entire time window
9: Get mean voltage V^*^ and location C^*^ of them
10: **if** V^*^ ≥ 0.2 **then**
11: Initialize the translation range
12: **else**
13: Expand the translation range
14: **end if**
15: Set $T_{i}=C^{*}$
16: **end for**
17: **Return** *T*

## 3. Results

Since target candidates are randomly generated, the results of each track may differ for the same test sequence. So in this paper, we select the constant random seed in the test phase to ensure the target candidates generated for the same location are fixed. Our model is implemented by PyTorch (Paszke et al., 2019), and runs on a 64-core Intel Xeon Gold 5218 2.30GHz CPU and an NVIDIA 2080Ti GPU.

### 3.1. DVSOT21

Although various event-based tracking algorithms have emerged in recent years, most of them indicate the target location by distinguishing whether each pixel belongs to the target or the background. However, such methods neither fully utilize implicit associations among events nor extract environmental features around the target, hence they are easily disturbed by inevitable noise. To exploit the surroundings of targets for more robust features, we use bounding box-based tracking in our work. While there are some event-based tracking datasets available, a number of targets within them are too small to generate enough events, which is difficult to support the emission of spikes in the last layer of SNN. So in this paper, we propose an event-based dataset DVSOT21 for bounding box-based single object tracking, which contains few tiny targets. Instead of recording event from sensors and manually labeling bounding boxes, the ESIM simulator (Rebecq et al., 2018) is applied to generate nine sequences with the spatial resolution of 640 × 480 pixels.

We design a new approach that implements the bounding boxes generation module to extend the ESIM simulator, getting the ground-truth bounding boxes of moving objects. First, the moving object is rendered separately to get grayscale images. Then the images are binarized and the contours are detected. Finally, circumscribed rectangles for the contours are produced as the ground-truth bounding boxes.

**Figure 5:**
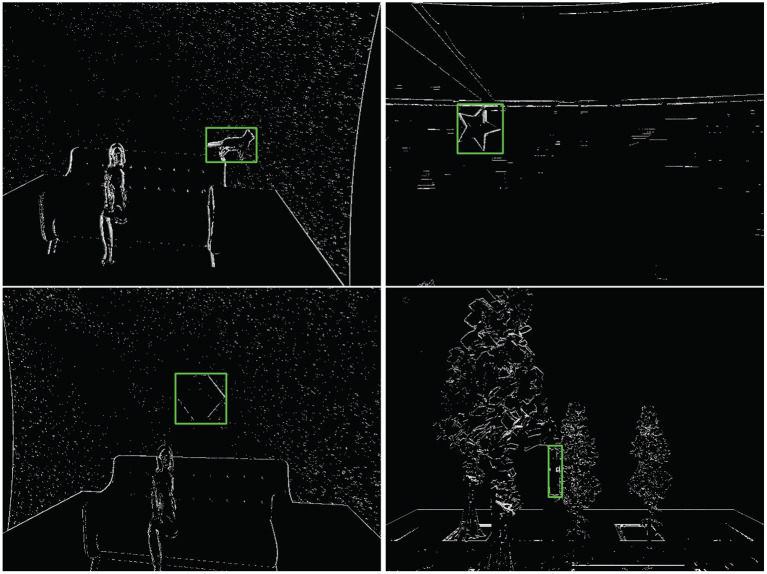
The first segment of four sequences in the DVSOT21 training set, where the green bounding boxes represent the corresponding ground-truths. From left to right and from top to bottom, the sequences are *woman*_*cat*, *trees*_*star*, *woman*_*cube* and *trees*_*bottle* respectively.

Each of the candidate scenes and moving objects are imported into a 3D computer graphics software, Blender (Community, 2018), from which the camera trajectories and object trajectories are generated and exported. To keep the camera field of view consistent in the Blender and ESIM simulator, we obtain the camera intrinsics matrix in the Blender. After the object models are imported and the config parameters are specified, the extended ESIM simulator outputs event-based sequences, and a brief description of these sequences is given in Table 1. We recorded four pairs of sequences and a single sequence. Since each pair of sequences has the same kind of moving objects, we use one for training and the other one for test. The single sequence in the test set is used to demonstrate that our algorithm is powerful enough to track objects even not appearing in the training set. Some segments in the DVSOT21 training set can be seen in Figure 5.

**Table 1:** The details of nine sequences on DVSOT21, where the top four sequences are used for training and the bottom five are used for test.

**Sequence Name**	**Challenges**	**Object**	**Background**
*woman*_*cat*	−	Cat	Galaxy
*trees*_*bottle*	−	Bottle	Mountain
*trees*_*star*	−	Star	City
*woman*_*cube*	−	Cube	Galaxy
*city*_*cat*	Fast moving + Rich texture	Cat	Sea
*city*_*bottle*	Partial occlusion + rich texture	Bottle	window
*city*_*star*	rotation + rich texture	star	window
*sky*_*cube*	Rotation + background clutter	Cube	Galaxy
*woman*_*ball*	Partial sparse events	Ball	Flower

### 3.2. Evaluation on DVSOT21

For DVSOT21, we collect all the events within 10ms as a segment, and we set the time resolution of SCTN to 1ms. Therefore, the time window of SCTN is equal to 10, while the input of each time step is different, which contains all the events in every 1ms. In this way, we take advantage of the temporal information in the events, reflecting the superiority of SNN.

We use *A* and *R* as evaluation metrics, which show accuracy and robustness of tracking and they are calculated as:

(8) $$\begin{array}{l} A=\overset{N^{seq}}{\underset{i=1}{\sum }}\overset{S_{i}}{\underset{j=1}{\sum }}\frac{O_{i,j}^{P}\cap O_{i,j}^{G}}{O_{i,j}^{P}\cup O_{i,j}^{G}} \end{array}$$

(9) $$\begin{array}{l} R=\overset{N^{seq}}{\underset{i=1}{\sum }}\overset{S_{i}}{\underset{j=1}{\sum }}Success_{i,j} \end{array}$$

where *N*^*seq*^ is the number of sequences and *S*_*i*_ is the number of segments in the i-th sequence. $O_{i,j}^{P}$ denotes the predicted bounding box in the j-th segment of the i-th sequence and $O_{i,j}^{G}$ denotes the corresponding ground-truth. *Success*_*i, j*_ has two values, 1 means tracking successfully in the j-th segment of the i-th sequence while 0 means failure. We will consider it as a failure case when the IoU between the predicted bounding box and the corresponding ground-truth is under 0.5. If a failure occurs, we will reinitialize the tracker in the next segment in order to better measure the tracking performance throughout the sequence.

Both *A* and *R* are important metrics to evaluate the performance of a tracker, but sometimes there will be inconsistency between A and R, i.e., a high *R* value with low *A* value or a low *R* value with high *A* value. Therefore, we need to define another metric in order to comprehensively evaluate the performance of a tracker. We use *ARscore* as follow:

(10) $$\begin{array}{l} ARscore=\left(1+\beta^{2}\right)\frac{AR}{\beta^{2}A+R} \end{array}$$

Such a calculation form is similar to F1score when β is set to 1. In our work, the low *R* value means that the tracker has been reinitialized many times, which will cause the *A* value to be falsely high, so we pay more attention to the *R* value by setting β = 2.

Table 2 illustrates the quantitative results of our method and some representative competing trackers on DVSOT21, where RCT (Delbruck, 2007) and our method are event-based trackers, and the others are conventional ANN-based trackers. In fact, GOTRUN (Held et al., 2016), SiamRPN (Li et al., 2018), SiamMask (Wang et al., 2019), Ocean (Zhang et al., 2020) and AutoMatch (Zhang et al., 2021) are all based on the Siamese Network, which has remarkable performance in object tracking. As for conventional trackers, we need to convert the input event stream into frames at first, and here we use the Adaptive Time-Surface with Linear Time Decay event-to-frame conversion algorithm in Chen et al. (2019). For all events generated every 10ms, they are expected to be converted into a frame. Above all, we need to subtract the timestamp corresponding to the earliest event in the event stream from the timestamps corresponding to all events to obtain t^*^. Then we can get the timestamp $t_{i}^{*}$ of the latest event *e*_*i*_ = (*x*_*i*_, *y*_*i*_, *p*_*i*_, *t*_*i*_) at the coordinates (*x*_*i*_, *y*_*i*_). So the pixel value of the frame can be calculated as follow:

(11) $$\begin{array}{l} F\left(x_{i},y_{i}\right)=round\left(\frac{255\times t_{i}^{*}}{10}\right) \end{array}$$

The pixel value of the locations where no event is produced are set to 0.

**Table 2:** Quantitative results on the five event-based test sequences from DVSOT21 dataset.

**Method**	***city***_***cat***		***city***_***bottle***		***city***_***star***		***sky***_***cube***		***woman***_***ball***		***all***	
	**A**	**R**	**A**	**R**	**A**	**R**	**A**	**R**	**A**	**R**	**A**	**R**
	**ARscore**		**ARscore**		**ARscore**		**ARscore**		**ARscore**		**ARscore**	
**RCT** (Delbruck, 2007)	0.054	0.020	0.014	0.014	0.344	0.130	0.435	0.294	0.114	0.102	0.223	0.135
	0.023		0.014		0.149		0.315		0.104		0.147	
**GOTURN** (Held et al., 2016)	0.511	0.490	0.643	0.890	0.532	0.554	0.597	0.773	0.522	0.636	0.562	0.681
	0.494		0.827		0.550		0.730		0.609		0.653	
**SiamRPN** (Li et al., 2018)	0.767	0.980	0.700	0.959	0.754	0.978	0.838	0.992	0.819	0.983	0.786	0.980
	0.928		0.893		0.923		0.956		0.945		0.934	
**SiamMask** (Wang et al., 2019)	0.749	0.980	0.721	0.973	0.775	0.957	0.814	1.000	0.863	0.992	0.797	0.982
	0.923		0.909		0.914		0.956		0.963		0.939	
**Ocean** (Zhang et al., 2020)	0.577	0.653	0.535	0.562	0.581	0.717	0.564	0.681	0.670	0.890	0.592	0.721
	0.636		0.556		0.685		0.654		0.835		0.691	
**AutoMatch** (Zhang et al., 2021)	0.631	0.755	0.620	0.795	0.661	0.880	0.682	0.891	0.728	0.932	0.674	0.869
	0.726		0.752		0.826		0.839		0.883		0.822	
**SCTN**	0.788	0.939	0.762	0.959	0.739	0.946	0.805	0.992	0.769	0.949	0.773	0.960
	0.904		0.912		0.896		0.948		0.907		0.916	

Red, blue and orange represent 1*st*, 2*nd* and 3*rd* respectively. Our method achieves competitive performance with a much simpler network compared to SiamRPN and SiamMask.

**Figure 6:**
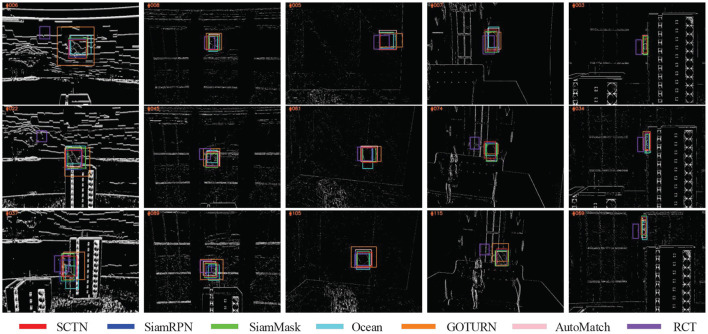
Qualitative results on the five event-based test sequences from DVSOT21 dataset, where the bounding box with red, blue, green, cayn, orange, pink and purple color represent SCTN, SiamRPN, SiamMask, Ocean, GOTURN, AutoMatch and RCT respectively. From left to right, the corresponding sequences are *city*_*cat*, *city*_*bottle*, *city*_*star*, *sky*_*cube* and *woman*_*ball* respectively.

SiamMask achieves the most outstanding performance, reaching 0.939 *ARscore* over five sequences. Besides, SiamRPN and our proposed method SCTN achieves the second and the third highest performance respectively, where SCTN even surpass SiamRPN and SiamMask on *city*_*bottle*. This is because SCTN focuses on the events generated by the movement of object contours rather than a gray-scale synthetic image patch, so it can accurately capture objects even if they are partially occluded. Furthermore, SCTN also has a relatively good performance on *woman*_*ball*, which means it can successfully track objects not appeared in the training set. In comparison, GOTURN, Ocean and AutoMatch usually achieve low *ARscore* value, due to the influence of rich texture, fast moving and motion blur. However, the performance of RCT is low. We find that RCT is mainly based on the clustering algorithm, so it is susceptible to noise events. Some qualitative results are presented in Figure 6.

### 3.3. Ablation experiments

To prove the importance of exponential IoU proposed in the loss function, we compare it with the loss function with linear IoU and the basic loss function. For the sake of fairness, the feedback of a sample with linear IoU in the loss function can be calculated as below:

(12) $$\begin{array}{l} R_{s}=\{\begin{array}{ll} \mu O_{s}-\nu , & s\in positive\;samples;\\ \;-0.3, & s\in negative\;samples. \end{array} \end{array}$$

where μ = 4 and ν = 2.5, mapping the values of positive feedback between (0.3, 1.5).

**Figure 7:**
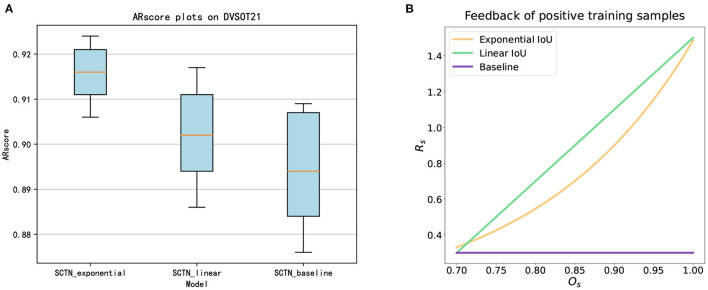
**(A)** ARscore plots of three models on DVSOT21, ranked the SCTN with exponential IoU in the loss function, the SCTN with linear IoU in the loss function and the SCTN with basic loss function in descending order of performance. **(B)** The feedback of positive training samples with three models.

The results of ablation experiments are shown in Figure 7A, which is calculated from the average of ten experiments. It can be seen that the SCTN with exponential IoU in the loss function is ranked top overall and its robustness is also the highest. Compared to the loss function with linear IoU, the loss function with exponential IoU is more discriminative. As illustrated in Figure 7B, for positive samples of different IoU, the feedback allocated by the loss function with exponential IoU is steeper. Besides, the SCTN with linear IoU in the loss function is superior to SCTN with basic loss function. The reason is that the basic loss function can only give constant feedback to samples. Nevertheless, the loss function with linear IoU is able to pay more attention to positive samples with larger IoU.

### 3.4. Energy consumption

To investigate the energy efficiency of the trained SCTN, we evaluate it on DVSOT21 and compare it to the ANN-based trackers. The energy consumption of ANN and SNN models can be calculated as follow:

(13) $$\begin{array}{l} E_{ANN}=n_{MAC}\times e_{MAC} \end{array}$$

(14) $$\begin{array}{l} E_{SNN}=n_{AC}\times e_{AC} \end{array}$$

where MAC denotes multiply-and-accumulate operation and AC denotes accumulate operation, *n* means the total number of operations, and *e* represents the energy cost per operation. As reported by (Han et al., 2015), a 32-bit floating point MAC and AC operation consume 4.6 pJ and 0.9 pJ in 45 nm technology respectively.

**Figure 8:**
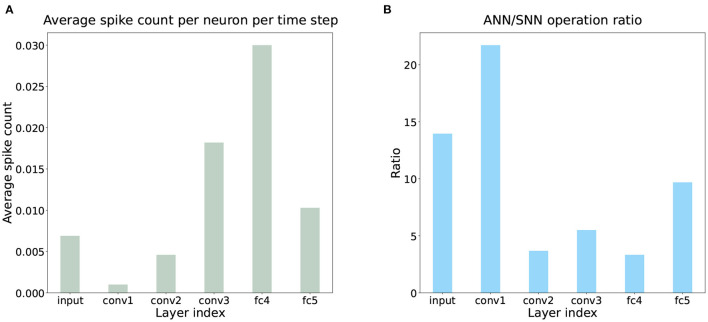
**(A)** Average spike count per neuron per time step of the trained SCTN on DVSOT21. It is shown that the neuronal activities of all network layers are sparse in the entire time window, which leads to the very low energy consumption of SCTN in Table 3. **(B)** The operation ratio of ANN/SNN in the same network structure.

We know that the energy consumed by SNN depends on the firing rate of spikes. As shown in Figure 8A, the spike firing rate of SCTN processing a segment is estimated by sampling 64 segments in DVSOT21 and calculating their average firing rates, which is very sparse across all network layers in the entire time window. But for ANN-based trackers, the energy consumption is a fixed number. As illustrated in Table 3, SiamRPN and SiamMask require 3203 and 8355 times total energy to SCTN. With a comparable tracking performance, SCTN can achieve energy-saving computing. In addition, Figure 8B shows that even in the same network structure, the energy consumption of ANN is much greater than that of SNN. However, the above calculation of energy consumption is actually incomplete, considering only synaptic operands. In fact, the movement of data between memory and CPU also has a certain energy consumption. This part of the energy consumption is related to the hardware environment, which is difficult to be quantified. Compared to the numerical operations in the GPU, it does not consume a large amount of energy consumption. Therefore, it does not have an impact on the comparison results of energy consumption, and SCTN still consumes much less energy than SiamRPN and SiamMask.

**Table 3:** Energy consumption of two ANN-based trackers and SCTN on processing a frame or a segment during tracking.

**Method**	**MAC/AC Ops**	**Energy consumption**
**SiamRPN**	4.82 × 10^9^	2.22 × 10^10^*pJ*
**SiamMask**	1.26 × 10^10^	5.79 × 10^10^*pJ*
**SCTN**	7.69 × 10^6^	6.93 × 10^6^*pJ*

### 3.5. Discussion

In fact, a general event-based object tracking algorithm can highlight the advantages of event cameras in many applications. Here, we discuss two major limitations of SCTN as below.

The first limitation is that SCTN cannot process the bounding boxes with spatial resolution less than 10 × 10. Because the number of events contained in the small bounding boxes is insufficient, the C-LIF neurons in the deep layers cannot emit spikes. Thus, investigating a more general model is necessary in the future.

The other limitation is that the tracking precision of our method is not as good as state-of-the-art ANN models. This is because SNN cannot deal with numeric regression problems directly, resulting in certain errors in generating target bounding boxes. Hence, an event based tracking model combining ANN and SNN is needed for the further work.

Thus, how to capture the tiny objects and improve the tracking performance of SNN is a worthwhile topic in the future. We believe this work could lay the foundation for building universal event-based object tracking on the neuromorphic hardware.

## 4. Conclusion

In this paper, we propose a novel spiking convolutional tracking network directly trained with SNN, which can process event stream without any other preprocessing operations. We propose a new loss function that introduces an exponential IoU in the voltage domain so as to make SCTN more suitable for object tracking. Moreover, we present a new publicly available event-based tracking dataset, dubbed DVSOT21. Experimental results on DVSOT21 demonstrate that our method achieves competitive performance with very low energy consumption compared to other competing trackers.

## Data availability statement

The raw data supporting the conclusions of this article will be made available by the authors, without undue reservation.

## Author contributions

MJ is responsible for coming up with ideas, doing experiments, and writing the article. ZW is responsible for recording the tracking dataset and writing the article. RY, QL, SX, and HT help revise the article and supplement experiments. All authors contributed to the article and approved the submitted version.

## References

[B1] Bertinetto L. Valmadre J. Henriques J. F. Vedaldi A. Torr P. H. (2016). Fully-convolutional siamese networks for object tracking, in European conference on computer vision (New York, NY: Springer), 850–865. 10.1007/978-3-319-48881-3_56

[B2] Chen H. Suter D. Wu Q. Wang H. (2020). “End-to-end learning of object motion estimation from retinal events for event-based object tracking. Proc. Innov. Appl. Artif. 34, 10534–10541. 10.1609/aaai.v34i07.6625

[B3] Chen H. Wu Q. Liang Y Gao X. Wang H. (2019). Asynchronous tracking-by-detection on adaptive time surfaces for event-based object tracking, in Proceedings of the 27th ACM International Conference on Multimedia (New York, NY: Association for Computing Machinery), 473–481. 10.1145/3343031.3350975

[B4] Chen X. Yan B. Zhu J. Wang D. Yang X. Lu H. (2021). Transformer tracking, in Proceedings of the IEEE/CVF Conference on Computer Vision and Pattern Recognition (CVPR) (IEEE), 8126–8135. 10.1109/CVPR46437.2021.00803

[B5] Community B. O. (2018). Blender - a 3D Modelling and Rendering Package. Amsterdam: Blender Foundation, Stichting Blender Foundation.

[B6] Cui Y. Cheng J. Wang L. Wu G. (2022). Mixformer: end-to-end tracking with iterative mixed attention. ArXiv abs/2203.11082. 10.1109/CVPR52688.2022.01324 38713562

[B7] Delbruck T. (2007). Jaer open source project. Retrieved. 10, 2014.

[B8] Gallego G. Delbruck T. Orchard G. M. Bartolozzi C. Taba B. Censi A.. (2020). Event-based vision: a survey, in IEEE Transactions on Pattern Analysis and Machine Intelligence. 10.1109/TPAMI.2020.3008413 32750812

[B9] Gerstner W. Kistler W. M. (2002). Spiking Neuron Models: Single Neurons, Populations, Plasticity. Cambridge, United Kingdom: Cambridge University Press. 10.1017/CBO9780511815706

[B10] Ghosh-Dastidar S. Adeli H. (2009). Spiking neural networks. Int. J. Neural Syst. 19, 295–308. 10.1142/S0129065709002002 19731402

[B11] Gu P. Xiao R. Pan G. Tang H. (2019). Stca: spatio-temporal credit assignment with delayed feedback in deep spiking neural networks. IJCAI 15, 1366–1372. 10.24963/ijcai.2019/189

[B12] Gütig R. (2016). Spiking neurons can discover predictive features by aggregate-label learning. Science. 351, 6277. 10.1126/science.aab4113 26941324

[B13] Han S. Pool J. Tran J. Dally W. (2015). Learning both weights and connections for efficient neural network, in Advances in Neural Information Processing Systems, 28.

[B14] Held D. Thrun S. Savarese S. (2016). Learning to track at 100 fps with deep regression networks, in European Conference on Computer Vision (New York, NY: Springer), 749–765. 10.1007/978-3-319-46448-0_45

[B15] Henriques J. F. Caseiro R. Martins P. Batista J. (2014). High-speed tracking with kernelized correlation filters. IEEE Trans. Pattern Anal. Mach. Intell. 37, 583–596. 10.1109/TPAMI.2014.2345390 26353263

[B16] Jiang R. Zhang J. Yan R. Tang H. (2021). Few-shot learning in spiking neural networks by multi-timescale optimization. Neur. Computat. 33, 2439–2472. 10.1162/neco_a_01423 34280263

[B17] Khoei M. A. Ieng S. H. Benosman R. (2019). Asynchronous event-based motion processing: from visual events to probabilistic sensory representation. Neur. Computat. 31, 1114–1138. 10.1162/neco_a_01191 30979350

[B18] Lagorce X. Meyer C. Ieng S.-H. Filliat D. Benosman R. (2014). Asynchronous event-based multikernel algorithm for high-speed visual features tracking. IEEE Trans. Pattern Anal. Mach. Intell. 26, 1710–1720. 10.1109/TNNLS.2014.2352401 25248193

[B19] Li B. Yan J. Wu W. Zhu Z. Hu X. (2018). High performance visual tracking with siamese region proposal network, in Proceedings of the IEEE Conference on Computer Vision and Pattern Recognition, 8971–8980. 10.1109/CVPR.2018.00935

[B20] Litzenberger M. Posch C. Bauer D. Belbachir A. N. Schon P. Kohn B.. (2006). Embedded vision system for real-time object tracking using an asynchronous transient vision sensor, in 2006 IEEE 12th Digital Signal Processing Workshop &4th IEEE Signal Processing Education Workshop (Teton National Park, WY: IEEE), 173–178. 10.1109/DSPWS.2006.265448

[B21] Luo Y. Xu M. Yuan C. Cao X. Zhang L. Xu Y.. (2021). Siamsnn: Siamese spiking neural networks for energy-efficient object tracking, in International Conference on Artificial Neural Networks (Springer), 182–194. 10.1007/978-3-030-86383-8_15

[B22] Mitrokhin A. Fermuller C. Parameshwara C. Aloimonos Y. (2018). Event-based moving object detection and tracking, in 2018 IEEE/RSJ International Conference on Intelligent Robots and Systems (IROS) (Madrid: IEEE), 1–9. 10.1109/IROS.2018.8593805

[B23] Nam H. Han B. (2016). Learning multi-domain convolutional neural networks for visual tracking, in Proceedings of the IEEE Conference on Computer Vision and Pattern Recognition, 4293–4302. 10.1109/CVPR.2016.465

[B24] Ni Z. Ieng S.-H. Posch C. Régnier S. Benosman R. (2015). Visual tracking using neuromorphic asynchronous event-based cameras. Neur. Computat. 27, 925–953. 10.1162/NECO_a_00720 25710087

[B25] Paszke A. Gross S. Massa F. Lerer A. Bradbury J. Chanan G.. (2019). Pytorch: An imperative style, high-performance deep learning library, in Neural Information Processing Systems (Vancouver, BC: Vancouver Convention Center; MIT Press).

[B26] Rebecq H. Gehrig D. Scaramuzza D. (2018). Esim: an open event camera simulator, in Conference on Robot Learning, 969–982.

[B27] Wang N. Zhou W. Wang J. Li H. (2021). Transformer meets tracker: Exploiting temporal context for robust visual tracking, in Proceedings of the IEEE/CVF Conference on Computer Vision and Pattern Recognition (CVPR) (IEEE), 1571–1580. 10.1109/CVPR46437.2021.00162

[B28] Wang Q. Zhang L. Bertinetto L. Hu W. Torr P. H. (2019). Fast online object tracking and segmentation: a unifying approach, in Proceedings of the IEEE/CVF Conference on Computer Vision and Pattern Recognition (Long Beach, CA: IEEE), 1328–1338. 10.1109/CVPR.2019.00142

[B29] Zhang Z. Liu Y. Wang X. Li B. Hu W. (2021). Learn to match: Automatic matching network design for visual tracking, in Proceedings of the IEEE/CVF International Conference on Computer Vision (IEEE), 13339–13348. 10.1109/ICCV48922.2021.01309

[B30] Zhang Z. Peng H. Fu J. Li B. Hu W. (2020). Ocean: Object-aware anchor-free tracking, in Computer Vision-ECCV 2020: 16th European Conference, Glasgow, UK, August 23-28, 2020, Proceedings, Part XXI 16 (New York, NY: Springer), 771–787. 10.1007/978-3-030-58589-1_46

