# Corrigendum: SCTN: event-based object tracking with energy-efficient deep convolutional spiking neural networks

**DOI:** 10.3389/fnins.2023.1204334

**Published:** 2023-05-16

**Authors:** Mingcheng Ji, Ziling Wang, Rui Yan, Qingjie Liu, Shu Xu, Huajin Tang

**Affiliations:** ^1^College of Computer Science and Technology, Zhejiang University, Hangzhou, China; ^2^College of Computer Science, Zhejiang University of Technology, Hangzhou, China; ^3^Machine Intelligence Laboratory, China Nanhu Academy of Electronics and Information Technology, Jiaxing, China; ^4^Zhejiang Lab, Hangzhou, China

**Keywords:** spiking neural networks, event cameras, object tracking, exponential IoU, event-based tracking dataset

In the published article, there was an error in the **Funding** statement that we omitted the acknowledgment information in this article. We would like to add this acknowledgment after the conclusion section. The correct funding statement appears below.

